# Fruit and Vegetable Production

**DOI:** 10.3390/plants12173125

**Published:** 2023-08-30

**Authors:** Lord Abbey, Mason MacDonald, Josephine Ampofo

**Affiliations:** 1 Department of Plant, Food, and Environmental Sciences, Faculty of Agriculture, Dalhousie University, Halifax, NS B2N 5E3, Canada; 2 Department of Food Science and Technology, University of California, Davis, CA 95616, USA; josephine.ampofo@mail.mcgill.ca

## 1. Background

Fruits and vegetables are generally known to contain important vitamins, fiber, essential minerals, and vital bioactive compounds that possess health benefits such as anti-inflammatory, antimicrobial, antioxidant, and anticancer properties [1]. These compounds can help protect against chronic diseases such as cancer, diabetes, and cardiovascular diseases. However, the composition of fruits and vegetables with regard to these compounds is dependent on plant genotypic, environmental, and management factors [2,3,4]. By nature, the production of fruits and vegetables requires high input and intensive management practices compared to other crops. As such, these crops are fragile and easily succumb to various environmental stressors during production and post-harvest processing, handling, and storage [5,6]. Consequently, collaboration between industry, researchers, and policymakers to develop or adopt novel technologies and techniques to improve fruit and vegetable crop growth, productivity, and harvest and edible qualities is paramount.

This Special Issue (Fruit and Vegetable Production) of the journal *Plants* focuses on the entire chain of fruit and vegetable production including post-harvest and marketing topics under field and greenhouse production systems. Therefore, it is not surprising that the information provided by this Special Issue will further strengthen the theory that effective collaboration between researchers and stakeholders across various food systems jurisdictions will continue to grow within these areas of research. This Special Issue comprises a collection of 12 peer-reviewed manuscripts covering basic and applied themes of crop response to organic amendments, biostimulants, irrigation, physiological stress, metabolic stimulation, and novel technologies for the efficient characterization of specialty crops, quality assessment, and cultivar evaluation.

## 2. The Main Findings of This Special Issue

### 2.1. Spatial and Compositional Variations in the Fruit Characteristics of Papaya (Carica papaya cv. Tainung No. 2) during Ripening

Chung et al. (2023) investigated selected compositional changes of papaya during ripening, with particular emphasis on nutrition and metabolite distributions between the calyx and stem end of the fruit [7]. From their findings, total carbohydrates, total protein, nitrogen, and potassium showed maximum accumulation at the stem end, with fructose, glucose, magnesium, and manganese accumulating at the calyx end [7].

### 2.2. Magnetically Treated Water in Phaseolus vulgaris L.: An Alternative Method to Develop Organic Farming in Cuba

“Magnetic biostimulation” or “magnetopriming” involves exposing water used for priming or irrigation to a magnetic field, with this technological approach proven to be effective with several growth-stimulating effects during germination and plant development [8]. Boix et al. (2023) investigated the effect of this technology on the potential of common beans in organic farming, with their work reporting the ability of magnetically treated water to increase germination, stem length, vigor, leaf area, seed weight, and chlorophyll and carbohydrate content [9].

### 2.3. The Growth and Biochemical Composition of Microgreens Grown in Different Formulated Soilless Media

Saleh et al. (2022) evaluated the effectiveness of four different growing media on four different plant species: namely, kale (*Brassica oleracea* L. var. acephala), Swiss chard (*Beta vulgaris* var. cicla), arugula (*Eruca vesicaria* ssp. sativa), and pak choi (*Brassica rapa* var. chinensis) grown as microgreens. Two media were identified as having the most beneficial effect on microgreens: (1) 30% vermicast + 30% sawdust + 10% perlite + 30% PittMoss and (2) 30% vermicast + 20% sawdust + 20% perlite + 30% mushroom compost [10]. Each of the above growing media consistently increased the content of chlorophyll, carotenoids, phenolics, and flavonoids and increased antioxidant enzyme activity. The second of the above treatments also significantly increased yields [10].

### 2.4. Irrigation Effect on the Yield, Skin Blemishes, Phellem Formation, and Total Phenolics of Red Potatoes

Jiang et al. (2022) investigated the benefits of irrigation on Dark Red Norland potatoes known to be drought sensitive. The use of irrigation improved yields by 20.6% and reduced surface cracking by 48.5% [11]. Microscopy imaging analysis demonstrated that tubers from the rainfed trials formed higher numbers of suberized cell layers than those of the irrigated potatoes. In addition, surface cracking and silver patch skins had more suberized cell layers than the normal skins. The irrigated potatoes also had a significantly higher concentration of total polyphenols [11].

### 2.5. Changes in Soil Characteristics, Microbial Metabolic Pathways, TCA Cycle Metabolites, and Crop Productivity following the Frequent Application of Municipal Solid Waste Compost

The use of municipal solid waste compost is well established, but Abbey et al. (2022) investigated the effect of long-term changes in soil microbial composition and subsequent translation on plant metabolic pathways. Both annual and biennial applications of municipal compost increased yields, with annual application being the most effective technique [12]. Furthermore, the same effect was found with increased microbial function and tricarboxylic metabolites, thus suggesting the potential application of municipal compost as a novel long-term green solution for improving soil health [12].

### 2.6. Changes in the Biogenic Amines of Two Table Grapes (cv. Bronx Seedless and Italia) during Berry Development and Ripening

Incesu et al. (2022) studied the accumulation of biogenic amines in two varieties of grape berries during ripening. Although a difference in biogenic amine concentration between the varieties was observed, there was a consistent increase in concentration throughout the ripening process [13]. The concentration of most of the biogenic amines analyzed increased linearly from the beginning of berry touch to when the berries ripened during the harvesting stage. The most common biogenic amine in the grape varieties was putrescine, followed by histamine, agmatine, and tyramine.

### 2.7. The Field Application of a Vis/NIR Hyperspectral Imaging System for the Non-Destructive Evaluation of the Physicochemical Properties of ‘Madoka’ Peaches

One major challenge in assessing the physiological and biochemical characteristics of fruits and vegetables is the destructive approaches available for quality assessment. To help solve this problem, Jang et al. (2022) evaluated a vis/NIR hyperspectral imaging system as a non-destructive quality assessment method in peaches [14]. The coefficient of determination was higher than 0.8 for chromaticity and soluble solids content, although it was slightly lower (0.6 to 0.7) for firmness and titratable acidity. Their study demonstrates the potential of hyperspectral imaging as a method for the non-destructive estimation of specific quality characteristics, and as such, it would be interesting to investigate its use for other parameters.

### 2.8. Cultivar and Post-harvest Storage Duration Influence Fruit Quality and the Nutritional and Phytochemical Profiles of Soilless-Grown Cantaloupe and Honeydew Melons

Pulela et al. (2022) contrasted differences between honeydew melons and cantaloupes grown using Hygromix^®^ growing media covered in a layer of vermiculite. The accumulated concentrations of vitamin C, β-carotene, zinc, phosphorous, potassium, magnesium, and calcium were all higher in the cantaloupes studied [15]. In general, total soluble solids, color intensity, pH, and flavonoids all increased during storage while vitamin C, flavonoids, and phenolics all decreased [15].

### 2.9. Maturation and the Post-Harvest Resting of Fruits Affect the Macronutrient and Protein Content of Sweet Pepper Seeds

Colombari et al. (2022) harvested sweet pepper fruits prior to physiological maturity in order to investigate the seed changes that occur during post-harvest resting (i.e., the time period between harvest and seed collection). The key findings of the study were that potassium, calcium, and magnesium decreased during fruit maturation while seed albumin, globulin, prolamin, and glutelin all increased [16].

### 2.10. The Effect of Pyroligneous Acid on the Productivity and Nutritional Quality of Greenhouse Tomato

Pyroligneous acid is a liquid obtained from the condensation of smoke from biodiesel or biochar formation and has been shown to be a potent biostimulant. Ofoe et al. (2022) applied concentrations between 0 and 2% as a root soak to tomato plants, which increased the number and mass of the tomato fruits [17]. The concentrations of several bioactive compounds such as total phenolics, flavonoids, and ascorbate were also improved by pyroligneous acid, as well as total dissolved solids [17]. It was shown through this study that pyroligneous acid could play a role in greenhouse tomato production.

### 2.11. Plant Growth-Promoting Rhizobacteria Improve the Growth and Fruit Quality of Cucumber under Greenhouse Conditions

Zapata-Sifuentes et al. (2022) studied the effect of three rhizobacteria species on cucumber plant growth in the hopes of reducing dependency on chemical fertilizer inputs. All three investigated species demonstrated improved cucumber growth parameters such as plant height, fruit length, and yield [18]. The researchers also observed significant accumulated levels of phenolics, flavonoids, and vitamin C and improved antioxidant capacity [18]. From the results of their study, it was found that rhizobacteria have the potential to improve both the quantity and quality of cucumbers.

### 2.12. Ascorbic Acid’s Preconditioning Effect on Broccoli Seedling Growth and Photosynthesis under Drought Stress

MacDonald et al. (2022) explored the use of ascorbic acid as a seed priming agent in broccoli following previous success in other species. Broccoli was not grown to harvest in this experiment, but there were increases in critical parameters such as plant growth, photosynthesis, water use efficiency, leaf area, and membrane protection [19]. Perhaps more interesting is that these same critical parameters also increased during drought stress, pointing to ascorbic acid having a role in increased plant growth and drought stress mitigation.
